# Persistence of oncogenic and non-oncogenic human papillomavirus is associated with human immunodeficiency virus infection in Kenyan women

**DOI:** 10.1177/2050312120945138

**Published:** 2020-07-28

**Authors:** Yan Tong, Philip Tonui, Aaron Ermel, Omenge Orang’o, Nelson Wong, Maina Titus, Stephen Kiptoo, Kapten Muthoka, Patrick J Loehrer, Darron R Brown

**Affiliations:** 1 Indiana University School of Medicine, Indianapolis, IN, USA; 2 Moi University, Eldoret, Kenya; 3 Maseno University, Maseno, Kenya

**Keywords:** Infectious diseases, oncology, women’s health

## Abstract

**Objectives:**

Cervical cancer is caused by persistent infection with oncogenic, or “high-risk” types of human papillomaviruses, and is the most common malignancy in Kenyan women. A longitudinal study was initiated to investigate factors associated with persistent human papillomavirus detection among HIV-infected and HIV-uninfected Kenyan women without evidence of cervical dysplasia.

**Methods:**

Demographic/behavioral data and cervical swabs were collected from HIV-uninfected women (n = 82) and HIV-infected women (n = 101) at enrollment and annually for 2 years. Human papillomavirus typing was performed on swabs (Roche Linear Array). Logistic regression models of human papillomavirus persistence were adjusted for demographic and behavioral characteristics.

**Results:**

HIV-infected women were older and less likely to be married and to own a home and had more lifetime sexual partners than HIV-uninfected women. All HIV-infected women were receiving anti-retroviral therapy at enrollment and had satisfactory CD4 cell counts and HIV viral loads. One- and two-year persistent human papillomavirus detection was significantly associated with HIV infection for any human papillomavirus, high-risk human papillomavirus, International Agency for the Research on Cancer-classified high-risk human papillomavirus, and non-oncogenic “low-risk” human papillomavirus.

**Conclusion:**

Persistent detection of oncogenic and non-oncogenic human papillomavirus was strongly associated with HIV infection in Kenyan women with re-constituted immune systems based on satisfactory CD4 cell counts. In addition to HIV infection, factors associated with an increased risk of human papillomavirus persistence included a higher number of lifetime sex partners. Factors associated with decreased risk of human papillomavirus persistence included older age and being married. Further studies are needed to identify the immunological defects in HIV-infected women that allow human papillomavirus persistence, even in women receiving effective anti-retroviral therapy. Further studies are also needed to determine the significance of low-risk human papillomavirus persistence in HIV-infected women.

## Background

Cervical cancer is responsible for nearly 300,000 deaths annually worldwide; 90% occur among women living in low- and middle-income countries.^1–3^ Cervical cancer is the most common malignancy in women living in sub-Saharan Africa.^4–6^ The incidence and mortality rate (15 and 12 per 100,000 women per year, respectively) of cervical cancer in Kenya far exceed the rates for women living in the United States (4 and 1 per 100,000 women per year, respectively).^7^ Oncogenic types of human papillomaviruses (“high-risk” or HR-HPV) are the causative agents of cervical cancer.^8^ HR-HPV infections are common, but only a small percentage of women will develop cervical cancer; persistent detection of HR-HPV over a 1- or 2-year period is associated with an increased risk of cancer.^9–13^ In addition, women who are HIV-infected have a higher incidence of HR-HPV infection, pre-cancerous cervical lesions, and cervical cancer compared to HIV-uninfected women.^14–25^ Although progress is being made, the HIV epidemic continues in Kenya with a prevalence of approximately 6.9% among women aged 15 to 64 years.^26^

In spite of advances in cervical cancer screening and HPV vaccine development, many questions about HPV in HIV-infected women remain unanswered. In contrast to the decline of other HIV-associated cancers (Kaposi sarcoma and B-cell lymphoma) since anti-retroviral therapy (ART) became available, the incidence of cervical cancer has not declined.^27,28^ Several studies indicate that ART reduces HR-HPV detection and persistence, but other studies show limited or no benefit.^24,29–37^ Other knowledge gaps involving HIV-infected women include the significance of persistence of HR-HPV types other than HPV 16 and HPV 18, and the potential contributory role of “low-risk” (LR-HPV) types to cervical cancer. In addition, while HIV-induced immunosuppression accounts for much of the increased incidence of cervical, other co-factors may be important.

A previous analysis of this cohort at study enrollment showed that HR-HPV types were more frequently detected in HIV-infected women compared to HIV-uninfected women.^25^ A longitudinal study was, therefore, conducted to identify potentially modifiable behavioral and biological factors associated with persistence.

## Methods

### Ethical considerations

Study approval was granted from the Moi Teaching Referral Hospital (MTRH) and Moi University School of Medicine, Eldoret, Kenya, the Kenya Medical Research Institute’s Scientific and Ethics Review Unit (KEMRI-SERU), and the Institutional Review Board of Indiana University School of Medicine. All study participants received a written copy of the consent (English or Swahili).

### Nature of study

This was a prospective cohort study conducted in Eldoret, Kenya, to identify potentially modifiable behavioral and biological factors associated with persistence of HPV in HIV-infected and HIV-uninfected women. These women were recruited at the Academic Model Providing Access to Healthcare (AMPATH) Cervical Cancer Screening Program at MTRH.^25,38^

### Study participants

A balanced (HIV-infected/-uninfected) cohort of women aged 18 to 45 years living within 30 km of Eldoret was recruited. These women had no evidence of cervical disease based on visual inspection with acetic acid (VIA) at enrollment. An enrollment of 220 participants was planned. To achieve a balance of 50% HIV-infected and 50% HIV-uninfected, women with normal VIA examinations were invited to participate in a sequential manner. Because approximately 60%–65% of women presenting to the clinic have been HIV-infected in recent years, it was expected that the goal of 110 HIV-infected women would be reached before the enrollment of 110 HIV-uninfected women. Therefore, when 110 HIV-infected women were enrolled, approximately 90 HIV-uninfected women were enrolled, and only HIV-uninfected women were invited to participate from that point on until the planned 110 HIV-uninfected were enrolled. However, due to several extra women being enrolled, some women eventually not agreeing to participate, and some women eventually being excluded, the final overall study included 106 HIV-uninfected and 116 HIV-infected women.^25^

For inclusion into this analysis of HPV persistence, a participant’s enrollment cervical swab and at least one swab from the Year 1 or Year 2 visits had to be available. A total number of 183 women, including 82 HIV-uninfected and 101 HIV-infected women, had such swabs performed and were therefore included in the analysis.

Exclusion criteria included abnormal VIA, a history of an abnormal VIA or Pap smear, previous diagnosis of cervical intraepithelial lesion (CIN) or cervical cancer, signs or symptoms of *Chlamydia trachomatis* or *Neisseria gonorrhoeae*, current pregnancy, inability to consent, or medical illness rendering the participant unable to attend visits. Participants were provided phone credit to contact study nurses if needed and were compensated for their time and effort at each visit as described.^25^

### Sample size calculation

The sample size of this study was chosen to ensure sufficient power to address the specific research questions. It was expected that approximately 60% of HIV-infected women and 40% of HIV-uninfected women would have HPV detected in annually collected cervical swabs during the longitudinal study. The actual HPV detections in our study sample was 59.1% among HIV-infected women and 35.2% among HIV-uninfected women at enrollment.^25^ In addition, we had expected persistent HPV detections during the longitudinal follow-up would be approximately 45% among HIV-infected women and 20% among HIV-uninfected women. Our sample size of 222 participants provided 85% power to detect a 20% difference in HPV detection and 98% power to detect a 25% difference in persistent HPV detection between HIV-infected and HIV-uninfected women based on chi-square tests at a two-sided 0.05 significance level. Despite loss of follow-ups in the longitudinal study, our analytical sample size of 183 women (101 HIV-infected women and 82 HIV-uninfected women) still provided a 95% power to detect a 25% difference in persistent HPV detection between HIV-infected and HIV-uninfected women.

### Interview, data, and sample collection

Structured face-to-face interviews by trained researchers were conducted at enrollment as previously described, and a brief questionnaire was utilized to gather certain demographic and behavioral information.^25^ Women were followed for 2 years with clinic visits every 12 months for this analysis. At enrollment, Year 1, and Year 2, a nurse or physician collected a cervical swab for HPV testing as part of the pelvic examination. Swabs were placed in standard transport media then frozen at −80°C. For HIV-infected women, variables were collected from the AMPATH Medical Record System including date of HIV diagnosis, ART use, HIV viral loads, and CD4 cell counts.

Per Kenya standard of care, all women were asked to return annually for VIA in the AMPATH Clinic. There are no guidelines in Kenya for specific treatment related to HR-HPV detection, whether incident or persistent, so no specific changes in treatment were recommended based on HPV findings.

### HPV testing

Specimens were transported to the Kenya Medical Research Institute–University of Massachusetts Medical School laboratory for processing as previously described.^25^ The Roche Linear Array (Roche Molecular Systems, Inc., Branchburg, NJ, USA) was used to determine HPV types as previously described.^39^ For the purpose of analysis, the 37 HPV types detected in the Roche Linear Array Assay were divided into four groups: Any (all) HPV types, HR-HPV, International Agency for the Research on Cancer (IARC) HR-HPV types, as designated by the IARC,^40^ or LR-HPV. Individual types assigned to these groups are shown at the bottom of Table 6.

### Definition of persistent HPV detection

Persistent HPV detection was defined in two ways: first, at the level of type-specific HPV records and second, to confirm the results of the first analysis and to provide an analysis in a clinically relevant format at the level of study participants. At the level of type-specific persistent HPV detection records, four “detection patterns” for each type-specific HPV in swabs were defined:

Pattern 1: no type-specific HPV detection in swabs at enrollment or follow-up visits.
Pattern 2: Non-persistent detection: a positive type-specific HPV detection in only one swab sample.
Pattern 3: 1-year persistent detection: a positive type-specific HPV detection in two consecutive swabs.
Pattern 4: 2-year persistent detection: a positive type-specific HPV detection in swabs obtained 2 years apart.

Hypothetical examples illustrate how persistence was determined at the type-specific persistent detection level. A participant may have had HPV 16 detected at enrollment, but not at Year 1 or Year 2. This would represent non-persistent detection (Pattern 2) for HPV 16. In addition, she may have had HPV 45 detected at all three visits, representing a 2-year persistent detection (Pattern 4) for HPV 45. If no other HPV types were detected, these types would each be assigned “no detection” (Pattern 1). Altogether there were 183 women × 37 HPV types = 6771 type-specific persistent HPV detection records (3034 records from HIV-uninfected women and 3737 from HIV-infected women).

Persistent HPV detection was also defined at the level of study participants, defined as the highest level of persistent detection pattern in the descending order of “2-year persistent detection,” “1-year persistent detection,” “non-persistent detection,” and “no detection” among a woman’s type-specific HPV detection records within a group (Any HPV, HR-HPV, IARC HR-HPV, or LR-HPV). For example, a participant may have had HPV types 18 and 33 detected at enrollment, and HPV 33 at Year 1 but not Year 2. No other HPV types were detected. This participant thus had “1-year persistent detection” for HPV 33 and “non-persistent detection” for HPV 18, and she would be classified as “1-year persistent detection” for any HPV types, HR-HPV types, and IARC HR-HPV types due to her highest persistent detection level from HPV 33. For LR-HPV types, she would be classified as “no detection.”

### Statistical analysis

Demographic and behavioral characteristics of participants at enrollment (age, marital status, educational level, home ownership, walking distance to health care of ⩾60 min, number of lifetime sex partners, and age of first sex) were summarized by descriptive statistics and compared between HIV-infected/uninfected women using t-tests, chi-square tests, or Wilcoxon rank sum tests. CD4 counts and HIV viral loads at enrollment and follow-up visits were reported for HIV-infected women. Patterns of “1-year persistent detection” and “2-year persistent detection” were then combined together as “persistent detection” for subsequent analysis. Counts and percentages of the five most frequently detected HR- and LR-HPV types in HIV-infected and HIV-uninfected women were reported.

Logistic regression models were fit to examine the associations between overall HPV detection (“non-persistent detection” and/or “1-year persistent detection” and/or “2-year persistent detection” vs “no detection”) and HIV status at the type-specific detection records level and at the study participant level separately. Furthermore, logistic regression models were fit to examine associations between persistent HPV detection (“1-year persistent detection” and/or “2-year persistent detection” vs “no detection” and/or “non-persistent detection”) and HIV status at the type-specific detection records level and at the study participant levels separately. The generalized estimating equation method was used in the regression models at the level of type-specific detection records to account for the correlations between multiple records from the same participant.

In addition, associations of CD4 cell counts with HPV persistence were examined among HIV-infected women using logistic regression models performed at the level of study participants. HIV-infected women were classified into four categories based on CD4 counts at enrollment: ⩽200, 201–350, 351–500, and >500 cells/µL. The risk of persistent HPV detection (1-year persistent and/or 2-year persistent detections) was compared between women who had the lowest CD4 counts (⩽200 cells/µL) with women who had higher CD4 counts. Demographic and behavioral characteristics were included in all logistic regression models as potential confounders. Analyses were performed using SAS Version 9.4 (Cary, NC, USA).

## Results

### Characteristics of participants

Of the 82 HIV-uninfected women, 58 (70.7%) attended all three visits, 8 (9.8%) attended the Enrollment plus Year 1 visits, and 16 (19.5%) attended the Enrollment plus Year 2 visits. For the 101 HIV-infected women, 77 (76.2%) attended all three visits, 15 (14.9%) attended the Enrollment plus Year 1 visits, and 9 (8.9%) attended the Enrollment plus Year 2 visits (p = 0.089).

A comparison of women in the analytic sample (n = 183) and women excluded due to loss of follow-up (n = 39) showed that women excluded were younger than the remaining women (32.0 vs 37.0 years, p < 0.001), but no significant differences were found in HIV status, marital status, education level, home ownership, walking distance to health care, number of lifetime sexual partners, or age of first sexual experience. Compared to HIV-uninfected women, HIV-infected women were significantly older and less likely to be married or to own a home, had more lifetime sexual partners, and had a lower age of first sexual intercourse (Table 1).

**Table 1.** Characteristics of study participants at enrollment (n = 183).

Characteristics	Overall (n = 183)	HIV-uninfected (n = 82)	HIV-infected (n = 101)	p-value
Median age (IQR)	37.0 (31.0–41.0)	34.0 (30.0–38.0)	38.0 (34.0–41.0)	0.003^a^
Married, n (%)	93 (50.8%)	59 (72.0%)	34 (33.7%)	<0.001^b^
More than secondary school education, n (%)	21 (11.5%)	12 (14.6%)	9 (8.9%)	0.134^b^
Home ownership, n (%)	41 (22.4%)	24 (29.3%)	17 (16.8%)	0.047^b^
Walking distance to health care ⩾60 min, n (%)	24 (13.1%)	10 (12.2%)	14 (13.9%)	0.579^b^
Median number of lifetime sex partners (IQR)	3.0 (2.0–5.0)	3.0 (1.0–4.0)	3.0 (3.0–6.0)	<0.001^c^
Median age of first sex (IQR)	17.0 (16.0–20.0)	18.0 (16.0–20.0)	17.0 (15.0–19.0)	0.027^a^

IQR: interquartile range.

a p-value from t-test.

b p-value from chi-square test.

c p-value from Wilcoxon sum rank test.

### HIV-specific characteristics

All 101 HIV-infected women were receiving ART at enrollment. The initiation date of ART was available in medical records for 93 of these 101 HIV-infected women; the mean duration of ART use for these 93 women was 4.3 years (Table 2). The median CD4 count at enrollment for 98 women with available results was 538 cells/µL (range 17–1474, interquartile range (IQR) 379–771). All HIV-infected women had HIV viral load measured at enrollment: 74 women (73.3%) had an undetectable HIV viral load (<40 copies/mL in the Roche Amplicor Assay). The median HIV viral load at enrollment for the 27 women with a detectable value was 5739 copies/mL (IQR 140–23,091). At Year 1, 88 of 92 HIV-infected women remaining in the study (95.7%) were receiving ART. For 90 participants with available results, the median CD4 count was 598 cells/µL (range 47–1470, IQR 438–715); 81 (90%) had an undetectable HIV viral load, and the median HIV viral load for the remaining 9 women was 4013 copies/mL (IQR 218–7081). At Year 2, 84 of 86 HIV-infected women (97.7%) remaining in the study were receiving ART. The median CD4 cell count for 60 women with available results was 620 cells/µL (range 164–1655, IQR 453–891). For the 77 women with available results, 73 (94.8%) had an undetectable HIV viral load; the median HIV viral load for the remaining 3 women was 90 copies/mL (IQR 48–5698).

**Table 2.** CD4 cell counts and HIV viral loads for HIV-infected women (n = 101 women).

Visit	No. of women in analysis	No. of women with available CD4 count	CD4 count (cells/µL) for women with available data				
			Median	25% percentile	75% percentile		
CD4 count (cells/µL)							
Enrollment	101	98	538	379	771		
Year 1	92	90	598	438	715		
Year 2	86	60	620	453	891		
Visit	No. of women in analysis	No. of women with available HIV viral load	No. of women with undetectable HIV viral load	No. of women with detectable HIV viral load	Viral load (copies/mL) for women with a detectable level		
					Median	25% percentile	75% percentile
HIV viral load (copies/mL)							
Enrollment	101	101	74	27	5739	140	23,091
Year 1	92	90	81	9	4013	218	7081
Year 2	86	77	73	4	90	48	5698

### HPV type detection in cervical swabs

For all cervical swabs from all women, >99% were adequate based on positive β-globin results. The most frequently detected HR-HPV types throughout the study were HPV 16, 51, 58, 59, and 66; the most frequently detected LR-HPV types were HPV 54, 61, 62, 83, and 84 (Table 3). For each of these individual HPV types, and for all other types detected in the Roche Linear Assay, non-persistent and persistent detection were generally higher among HIV-infected women compared to HIV-uninfected women, but these differences did not reach statistical significance for any individual HPV type (data not shown).

**Table 3.** The most frequently detected high-risk and low-risk HPV types among study participants by HIV status (n = 183 Women).

	HIV-uninfected women (n = 82)				HIV-infected women (n = 101)	
	Overall detection,^a^ n (%)	Non-persistent detection, n (%)	Persistent detection,^b^ n (%)	Overall detection,^a^ n (%)	Non-persistent detection, n (%)	Persistent detection,^b^ n (%)
High-risk						
HPV 16	6 (7.3)	4 (4.9)	2 (2.4)	14 (13.9)	10 (9.9)	4 (4.0)
HPV 51	4 (4.9)	4 (4.9)	0 (0.0)	12 (11.9)	5 (5.0)	7 (6.9)
HPV 58	8 (9.8)	6 (7.3)	2 (2.4)	16 (15.8)	10 (0.9)	6 (5.9)
HPV 59	4 (4.9)	4 (4.9)	0 (0.0)	13 (12.9)	12 (11.9)	1 (1.0)
HPV 66	1 (1.2)	1 (1.2)	0 (0.0)	14 (13.9)	6 (5.9)	9 (8.9)
Low-risk						
HPV 54	4 (4.9)	4 (4.9)	0 (0.0)	12 (11.9)	7 (6.9)	5 (5.0)
HPV 61	2 (2.4)	2 (2.4)	0 (0.0)	12 (11.9)	9 (8.9)	3 (3.0)
HPV 62	6 (7.3)	6 (7.3)	0 (0.0)	18 (17.8)	13 (12.9)	5 (5.0)
HPV 83	5 (6.1)	4 (4.9)	1 (1.2)	15 (14.9)	8 (7.9)	7 (6.9)
HPV 84	4 (4.9)	4 (4.9)	0 (0.0)	15 (14.9)	8 (7.9)	7 (6.9)

HPV: human papillomaviruses.

a Overall detection was defined as patterns of “non-persistent detection,” “1-year persistent detection,” and/or “2-year persistent detection.”

b Persistent detection was defined as patterns of “1-year persistent detection” and/or “2-year persistent detection.”

HPV detection patterns were analyzed by HIV status at the type-specific HPV detection records level and at the study participant level (Table 4). In the type-specific detection record-level analysis, any HPV, HR-HPV types, IARC HR-HPV, and LR-HPV as groups were all detected more often in HIV-infected women compared to HIV-uninfected women (Table 4). In addition, 1- and 2-year persistent detections in these groups were more frequent in HIV-infected women compared to HIV-uninfected women. In the analysis performed at the participant level, any HPV and LR-HPV groups were detected more often in HIV-infected women compared to HIV-uninfected women (Table 4). In addition, 1- and 2-year persistent detections in these groups were more frequent in HIV-infected women compared to HIV-uninfected women (Table 4). Detection and persistence of HR-HPV types and IARC HR-HPV were more frequent in HIV-infected women compared to HIV-uninfected women, but the differences did not reach statistical significance at the participant level (Table 4).

**Table 4.** HPV detection patterns by HIV status summarized at the type-specific HPV detection records level and at the study participant level.

HPV	Detection pattern	Type-specific detection records level data (n = 6771)^a^			Participant-level data (n = 183)		
		HIV-uninfected detection records (n = 3034), n (%)	HIV-infected detection records (n = 3737), n (%)	p-value^b^	HIV-uninfected participants (n = 82), n (%)	HIV-infected participants (n = 101), n (%)	p-value^b^
Any HPV^c^	No detection	2927 (96.5)	3475 (93.0)	<0.001	31 (37.8)	23 (22.8)	<0.001
	Non-persistent detection	91 (3.0)	176 (4.7)	–	37 (45.1)	29 (28.7)	–
	1-year persistent detection	11 (0.4)	59 (1.6)	–	10 (12.2)	28 (27.7)	–
	2-year persistent detection	5 (0.2)	27 (0.7)	–	4 (4.9)	21 (20.8)	–
HR-HPV^d^	No detection	1735 (96.2)	2075 (93.4)	<0.001	40 (48.8)	36 (35.6)	0.064
	Non-persistent detection	54 (3.0)	102 (4.6)	–	29 (35.4)	32 (31.7)	–
	1-year persistent detection	10 (0.6)	31 (1.4)	–	9 (11.0)	21 (20.8)	–
	2-year persistent detection	5 (0.3)	14 (0.6)	–	4 (4.9)	12 (11.9)	–
IARC HR-HPV^e^	No detection	1015 (95.2)	1207 (91.9)	0.005	45 (54.9)	42 (41.6)	0.072
	Non-persistent detection	40 (3.8)	70 (5.3)	–	27 (32.9)	31 (30.7)	–
	1-year persistent detection	6 (0.6)	24 (1.8)	–	6 (7.3)	17 (16.8)	–
	2-year persistent detection	5 (0.5)	12 (0.9)	–	4 (4.9)	11 (10.9)	–
LR-HPV^f^	No detection	1192 (96.9)	1400 (92.4)	<0.001	54 (65.9)	44 (43.6)	<0.001
	Non-persistent detection	37 (3.0)	74 (4.9)	–	27 (32.9)	26 (25.7)	–
	1-year persistent detection	1 (0.1)	28 (1.8)	–	1 (1.2)	19 (18.8)	–
	2-year persistent detection	0 (0.0)	13 (0.9)	–	0 (0.0)	12 (11.9)	–

HPV: human papillomaviruses; IARC: International Agency for the Research on Cancer.

a Each participant contributed for 37 type-specific HPV detection records, yielding 183 × 37 = 6771 total type-specific HPV detection records.

b p-value from chi-square test.

c Any HPV: HPV 6, 11, 16, 18, 26, 31, 33, 35, 39, 40, 42, 45, 51, 52, 53, 54, 55, 56, 58, 59, 61, 62, 64, 66, 67, 68, 69, 70, 71, 72, 73, 81, 82, 83, 84, CP6108, IS39.

d HR-HPV (high-risk HPV): HPV 16, 18, 26, 31, 33, 35, 39, 45, 51, 52, 53, 56, 58, 59, 66, 67, 68, 69, 70, 73, 82, IS39.

e IARC HR-HPV: HPV 16, 18, 31, 33, 35, 39, 45, 51, 52, 56, 58, 59, 66.

f LR-HPV (low-risk HPV): HPV 6, 11, 40, 42, 54, 55, 61, 62, 64, 71, 72, 81, 83, 84, CP6108.

### HIV status, demographic, and behavioral characteristics related to overall and persistent HPV detection

Logistic regression analysis was conducted to assess associations of overall HPV detection with HIV status and other characteristics of women in the longitudinal study (Table 5). In the analysis at the type-specific detection records level for overall detection (non-persistent and persistent), results from adjusted regression models demonstrated that any HPV type, HR-HPV, IARC-defined HR-HPV, and LR-HPV were detected significantly more frequently in HIV-infected women compared to HIV-uninfected women. Older age was associated with a significantly lower detection of any HPV, HR-HPV, and IARC HR-HPV, but not LR-HPV. A higher number of lifetime sex partners was associated with a significantly higher detection of any HPV, HR-HPV, and IARC HR-HPV. In the analysis at the participant level for overall (non-persistent and persistent) detection, results from adjusted regression models demonstrated that any HPV type, HR-HPV, IARC-defined HR-HPV, and LR-HPV were detected more frequently, but not significantly so, in HIV-infected women compared to HIV-uninfected women (Table 5). Older age was associated with a significantly lower detection of any HPV, HR-HPV, and IARC HR-HPV, but not LR-HPV. Being married was associated with a significantly lower detection of any HPV or LR-HPV.

**Table 5.** Logistic regression analysis of associations of overall HPV detection^a^ with HIV status and characteristics of women.

Variables	Any HPV^b^		HR-HPV^c^		IARC HR-HPV^d^		LR-HPV^e^	
	OR (95% CI)	p-value	OR (95% CI)	p-value	OR (95% CI)	p-value	OR (95% CI)	p-value
Analysis at the type-specific detection records level (n = 6771)								
HIV-infected	2.05 (1.41–3.00)	<0.001	1.93 (1.30–2.88)	0.001	1.91 (1.24–2.95)	0.003	2.25 (1.37–3.70)	0.001
Age	0.97 (0.94–0.99)	0.011	0.95 (0.93–0.98)	<0.001	0.95 (0.92–0.98)	0.001	0.99 (0.96–1.03)	0.638
Married	0.85 (0.59–1.22)	0.379	0.92 (0.62–1.37)	0.691	0.87 (0.57–1.32)	0.514	0.76 (0.48–1.18)	0.219
More than secondary school education	1.10 (0.63–1.94)	0.737	1.08 (0.60–1.94)	0.807	1.03 (0.54–1.96)	0.937	1.14 (0.58–2.25)	0.708
Home ownership	0.82 (0.51–1.33)	0.426	1.00 (0.63–1.59)	0.998	1.25 (0.76–2.04)	0.374	0.60 (0.31–1.19)	0.146
Walking distance to health care ⩾ 60 min	1.19 (0.83–1.73)	0.345	1.09 (0.72–1.67)	0.675	0.91 (0.57–1.47)	0.712	1.34 (0.81–2.23)	0.255
Number of lifetime sex partners	1.00 (1.00–1.01)	0.002	1.00 (1.00–1.01)	0.001	1.01 (1.00–1.01)	0.000	1.00 (1.00–1.01)	0.043
Age of first sex	1.00 (0.95–1.06)	0.950	0.99 (0.94–1.05)	0.806	1.02 (0.96–1.09)	0.443	1.01 (0.95–1.09)	0.672
Analysis at the participant level (n = 183)								
HIV-infected	2.08 (0.97–4.47)	0.061	1.89 (0.95–3.77)	0.071	1.83 (0.92–3.64)	0.083	1.94 (0.97–3.89)	0.062
Age	0.90 (0.84–0.97)	0.003	0.92 (0.87–0.97)	0.005	0.92 (0.87–0.97)	0.004	0.96 (0.91–1.02)	0.206
Married	0.37 (0.17–0.82)	0.015	0.76 (0.38–1.54)	0.445	0.61 (0.30–1.23)	0.164	0.45 (0.22–0.92)	0.028
More than secondary school education	1.41 (0.44–4.45)	0.562	0.97 (0.36–2.61)	0.954	0.98 (0.37–2.60)	0.960	2.65 (0.93–7.50)	0.067
Home ownership	1.96 (0.80–4.79)	0.140	1.41 (0.63–3.12)	0.401	1.81 (0.81–4.02)	0.146	0.64 (0.28–1.48)	0.300
Walking distance to health care ⩾ 60 min	1.27 (0.40–3.98)	0.683	0.79 (0.31–2.00)	0.620	0.83 (0.33–2.08)	0.686	1.52 (0.59–3.93)	0.387
Number of lifetime sex partners	1.07 (0.97–1.18)	0.196	1.05 (0.97–1.13)	0.217	1.05 (0.97–1.12)	0.209	1.05 (0.97–1.14)	0.204
Age of first sex	1.11 (0.99–1.25)	0.076	1.03 (0.93–1.14)	0.590	1.05 (0.95–1.17)	0.353	1.03 (0.92–1.15)	0.588

HPV: human papillomaviruses; IARC: International Agency for the Research on Cancer; OR: odds ratio; CI: confidence interval.

a Overall detection was defined as patterns of “non-persistent detection,” “1-year persistent detection” and/or “2-year persistent detection.”

b Any HPV: HPV 16,18, 26, 31, 33, 35, 39, 45, 51, 52, 53, 56, 58, 59, 66, 67, 68, 69, 70, 73, 82, IS39.

c HR-HPV (high-risk HPV): HPV 16, 18, 26, 31, 33, 35, 39, 45, 51, 52, 53, 56, 58, 59, 66, 67, 68, 69, 70, 73, 82, IS39.

d IARC HR-HPV: HPV 16, 18, 31, 33, 35, 39, 45, 51, 52, 56, 58, 59, 66.

e LR-HPV (low-risk HPV): HPV 6, 11, 40, 42, 54, 55, 61, 62, 64, 71, 72, 81, 83, 84, CP6108.

Logistic regression analysis of associations of persistent HPV detection (1 year and 2 year) with HIV status and characteristics of women was performed (Table 6). Analysis at the type-specific detection records level demonstrated that persistent detection of any HPV, HR-HPV, and IARC HR-HPV, and LR-HPV types were significantly higher in HIV-infected women compared to HIV-uninfected women. Older age was associated with lower persistent detection of any HPV, HR-HPV, and IARC HR-HPV, but not LR-HPV. A higher number of lifetime sex partners was associated with a higher detection of LR-HPV. Being married was significantly associated with a reduced risk of persistent detection of any IARC HR-HPV types. Analysis at the participant level also demonstrated that 1- and 2-year persistent detection of any HPV, HR-HPV, and IARC HR-HPV, and LR-HPV types were significantly higher in HIV-infected women compared to HIV-uninfected women. As was found in the analysis at the type-specific detection records level, being married was significantly associated with a significantly reduced risk of persistent detection of any IARC HR-HPV types.

**Table 6.** Logistic regression analysis of associations of persistent HPV detection^a^ with HIV status and characteristics of women.

Variables	Any HPV^b^		HR-HPV^c^		IARC HR-HPV^d^		LR-HPV^e^	
	OR (95% CI)	p-value	OR (95% CI)	p-value	OR (95% CI)	p-value	OR (95% CI)	p-value
Analysis at the type-specific detection records level (n = 6771)								
HIV-infected	4.01 (2.16–7.45)	<0.001	2.44 (1.29–4.63)	0.006	2.53 (1.25–5.12)	0.010	26.99 (3.58–203.66)	0.001
Age	0.97 (0.94–1.00)	0.033	0.94 (0.90–0.98)	0.003	0.94 (0.89–0.99)	0.012	1.01 (0.96–1.06)	0.769
Married	0.60 (0.33–1.10)	0.096	0.59 (0.32–1.11)	0.100	0.45 (0.22–0.92)	0.028	0.60 (0.22–1.58)	0.299
More than secondary school education	0.69 (0.30–1.56)	0.369	0.60 (0.22–1.70)	0.339	0.53 (0.16–1.81)	0.313	0.77 (0.21–2.85)	0.695
Home ownership	0.71 (0.35–1.48)	0.366	0.91 (0.42–1.99)	0.822	1.17 (0.50–2.75)	0.718	0.46 (0.14–1.52)	0.201
Walking distance to health care ⩾ 60 min	1.27 (0.74–2.17)	0.391	1.05 (0.54–2.02)	0.888	0.86 (0.36–2.02)	0.722	1.62 (0.72–3.64)	0.245
Number of lifetime sex partners	1.00 (1.00–1.01)	0.377	0.99 (0.97–1.01)	0.308	1.00 (0.98–1.01)	0.548	1.01 (1.00–1.01)	<0.001
Age of first sex	1.07 (0.98–1.16)	0.129	1.04 (0.94–1.16)	0.442	1.10 (0.97–1.24)	0.144	1.11 (0.99–1.24)	0.071
Analysis at the participant level (n = 183)								
HIV-infected	4.03 (1.85–8.76)	<0.001	2.71 (1.21–6.08)	0.016	2.72 (1.12–6.61)	0.028	26.96 (3.29–220.64)	0.002
Age	0.97 (0.91–1.03)	0.292	0.94 (0.89–1.00)	0.055	0.94 (0.88–1.00)	0.052	1.03 (0.95–1.11)	0.463
Married	0.51 (0.24–1.08)	0.078	0.54 (0.24–1.21)	0.137	0.36 (0.14–0.87)	0.024	0.35 (0.12–1.06)	0.063
More than secondary school education	0.91 (0.30–2.76)	0.861	0.70 (0.21–2.38)	0.567	0.56 (0.14–2.24)	0.414	1.49 (0.30–7.33)	0.626
Home ownership	0.89 (0.36–2.18)	0.797	1.01 (0.39–2.59)	0.989	1.29 (0.47–3.57)	0.620	0.61 (0.15–2.52)	0.497
Walking distance to health care ⩾ 60 min	1.19 (0.45–3.12)	0.730	0.97 (0.35–2.67)	0.953	0.67 (0.22–2.10)	0.494	1.60 (0.47–5.39)	0.448
Number of lifetime sex partners	1.02 (0.97–1.07)	0.379	0.98 (0.94–1.03)	0.534	0.99 (0.95–1.03)	0.647	1.03 (0.98–1.08)	0.302
Age of first sex	1.09 (0.97–1.23)	0.137	1.06 (0.93–1.20)	0.385	1.13 (0.99–1.29)	0.081	1.15 (0.96–1.37)	0.121

HPV: human papillomaviruses; IARC: International Agency for the Research on Cancer; OR: odds ratio; CI: confidence interval.

a Persistent detection was defined as patterns of “1-year persistent detection” and/or “2-year persistent detection.”

b Any HPV: HPV 16,18, 26, 31, 33, 35, 39, 45, 51, 52, 53, 56, 58, 59, 66, 67, 68, 69, 70, 73, 82, IS39.

c HR-HPV (high-risk HPV): HPV 16, 18, 26, 31, 33, 35, 39, 45, 51, 52, 53, 56, 58, 59, 66, 67, 68, 69, 70, 73, 82, IS39.

d IARC HR-HPV: HPV 16, 18, 31, 33, 35, 39, 45, 51, 52, 56, 58, 59, 66.

e LR-HPV (low-risk HPV): HPV 6, 11, 40, 42, 54, 55, 61, 62, 64, 71, 72, 81, 83, 84, CP6108.

### CD4 cell count and persistence HPV detection

The risk of persistent HPV detection (1- and/or 2-year persistent detections) was compared between HIV-infected women who had the lowest CD4 counts (⩽200 cells/µL) and those with higher CD4 counts. Regression models demonstrated that women with the lowest CD4 counts had a higher likelihood of HR-HPV persistence than women with higher CD4 counts (Table 7). As it was hypothesized that the increased HPV persistence occurred in spite of ART use and re-constituted immunological function (and was not simply due to low CD4 counts), an additional logistic regression analysis was performed after eliminating the nine women from the analysis with CD4 counts ⩽ 200 cells/µL. In this second analysis, HIV infection remained significantly associated with increased risk of persistent detection of any HPV (odds ratio (OR) 3.65, 95% CI 1.65–8.08, p = 0.001), HR-HPV (OR 2.36, 95% CI 1.03–5.42, p = 0.042), and LR-HPV (OR 22.38, 95% CI 2.70–185.48, p = 0.004); for IARC HR-HPV, the association was of borderline significance (OR 2.31, 95% CI 0.92–5.79, p = 0.073).

**Table 7.** Logistic regression analysis of associations of persistent HPV detection with CD4 counts (at enrollment) and characteristics of HIV-infected women (n = 98).

Variables	Any HPV^a^		HR-HPV^b^		IARC HR-HPV^c^		LR-HPV^d^	
	OR (95% CI)	p-value	OR (95% CI)	p-value	OR (95% CI)	p-value	OR (95% CI)	p-value
CD4 ⩽ 200 cells/µL (n = 9 women)	1	–	1	–	1	–	1	–
CD4 201–350 cells/µL (n = 13 women)	0.06 (0.00–0.72)	0.027	0.18 (0.02–1.48)	0.110	0.09 (0.01–0.93)	0.043	0.21 (0.03–1.65)	0.136
CD4 351–500 cells/µL (n = 17 women)	0.17 (0.01–1.92)	0.151	0.23 (0.03–1.62)	0.140	0.20 (0.03–1.44)	0.109	0.18 (0.03–1.27)	0.086
CD4 > 500 cells/µL (n = 59 women)	0.11 (0.01–1.02)	0.053	0.16 (0.03–0.91)	0.039	0.16 (0.03–0.89)	0.037	0.15 (0.03–0.86)	0.033
Age	1.00 (0.92–1.08)	0.906	0.94 (0.86–1.01)	0.109	0.93 (0.85–1.01)	0.086	1.06 (0.97–1.16)	0.198
Married	0.47 (0.17–1.33)	0.154	0.41 (0.13–1.28)	0.125	0.34 (0.09–1.21)	0.095	0.46 (0.14–1.50)	0.199
More than secondary school education	0.64 (0.13–3.17)	0.581	0.70 (0.12–4.02)	0.687	0.39 (0.04–3.66)	0.406	0.89 (0.14–5.56)	0.896
Home ownership	0.49 (0.13–1.92)	0.308	0.47 (0.10–2.16)	0.335	0.68 (0.14–3.28)	0.629	0.39 (0.08–2.01)	0.262
Walking distance to health care ⩾ 60 min	1.61 (0.45–5.81)	0.467	1.63 (0.44–5.95)	0.462	1.52 (0.39–5.93)	0.550	1.44 (0.38–5.37)	0.590
Number of lifetime sex partners	1.01 (0.98–1.05)	0.423	0.96 (0.89–1.04)	0.293	0.97 (0.89–1.05)	0.397	1.02 (0.98–1.07)	0.288
Age of first sex	1.13 (0.94–1.35)	0.199	1.02 (0.84–1.24)	0.836	1.06 (0.86–1.31)	0.589	1.16 (0.95–1.41)	0.136

HPV: human papillomaviruses; IARC: International Agency for the Research on Cancer; OR: odds ratio; CI: confidence interval.

a Any HPV: HPV 16,18, 26, 31, 33, 35, 39, 45, 51, 52, 53, 56, 58, 59, 66, 67, 68, 69, 70, 73, 82, IS39.

b HR-HPV (high-risk HPV): HPV 16, 18, 26, 31, 33, 35, 39, 45, 51, 52, 53, 56, 58, 59, 66, 67, 68, 69, 70, 73, 82, IS39.

c IARC HR-HPV: HPV 16, 18, 31, 33, 35, 39, 45, 51, 52, 56, 58, 59, 66.

d LR-HPV (low-risk HPV): HPV 6, 11, 40, 42, 54, 55, 61, 62, 64, 71, 72, 81, 83, 84, CP6108.

## Discussion

Most studies of HPV infection among sub-Saharan African women have been cross-sectional, indicating that HR- and LR-HPV infections are common and that HIV-infected women have a higher rate of HPV detection compared to HIV-uninfected women.^41–46^ This study confirmed that all types of HPV are common and detected more often in HIV-infected Kenyan women than in HIV-uninfected women. Most importantly, persistence of all groups of HPV (any HPV, HR-HPV, IARC HR-HPV types, and LR-HPV types) was associated with HIV infection. The ORs for HR-HPV persistence was approximately 2.5 for being HIV-infected and 27 for LR-HPV persistence, in spite of good adherence to ART and good immune function within the HIV-infected cohort. Even when those with the lowest CD4 counts were eliminated from the analysis, HIV-infected women still had significantly more HPV persistence than HIV-uninfected women. Because persistent HPV infections were so common in this cohort of HIV-infected women, most of whom were receiving ART and had “re-constituted” immune systems, it is likely that unidentified components of the immune system are necessary for control of HPV infections.

Few prior studies of HPV persistence in HIV-infected women have been conducted among women living in Africa. Adebamowo et al.^47^ found that 6-month persistent infections were more common in HIV-infected Nigerian women compared to HIV-uninfected women. ORs comparing HIV-infected and HIV-uninfected women were not significant for persistent LR-HPV detection. For HR-HPV, the OR was 4.49 (95% CI 2.26–8.91) for persistent detection. Some differences between this study and the study by Adebamowo et al. include a different HPV detection system (the SPF/LIPA system in the study by Adebamowo et al. vs the Roche Linear Array in this study), a shorter follow-up time in the study by Adebamowo et al. (6 vs 24 months), and a lack of ART data for the HIV-infected cohort in the study by Adebamowo et al. Mukanyangezi et al.^48^ studied HPV detection in 100 HIV-uninfected and 137 HIV-infected women living in Rwanda over a 24-month period. Persistent HR-HPV but not LR-HPV occurred more often among HIV-infected women than in HIV-uninfected women. It was not clear how persistence was defined, and no data on ART use were available.

Kelly et al.^49^ examined associations of HR-HPV detection and persistence with high-grade cervical intraepithelial neoplasia (CIN2+) in HIV-infected women living in Burkina Faso or South Africa. In contrast to this study, there was no HIV-uninfected control group of women. HPV typing of cervical swabs was performed at enrollment and at a second visit, 16 months later. Of 270 women living in Burkina Faso, 59.1% had 16-month persistence of a HR-HPV type, HPV 58 being the most frequently detected persistent type. Of 340 women living in South Africa, 44.7% had 16-month persistence of a HR-HPV type, HPV 35 being the most frequently detected persistent type. Persistence of any HR-HPV, HPV 16 plus HPV 18 combined, or HR types HPV 31, 33, 45, 52, and 58 combined were associated with development of CIN2+.

Several prior studies of HIV-infected women living in North America or Europe also indicate a high degree of HPV persistence, but as with the study by Kelly et al. mentioned above either did not include an HIV-uninfected control group or details of ART use were not reported. Konopnicki et al.^50^ assessed the impact of ART on persistent HR-HPV infection in 652 HIV-infected women living in Belgium, 84% of whom were from sub-Saharan Africa and 79% were receiving ART. HR-HPV was detected in 43% of women. Sustained HIV suppression and CD4 cell counts >500 cells/µL for >18 months were associated with decreased risk of persistent HR-HPV infection. Thorsteinsson et al.^13^ assessed risk factors of HR-HPV persistence among 71 HIV-infected women living in Denmark. Persistent HR-HPV detection occurred in 31 (43.7%) women. A CD4 count of <350 cells/μL predicted HR-HPV persistence. The ACTG A5029 trial followed 146 HIV-infected women prospectively after starting ART.^51^ Detection of most HPV types decreased over the 96 weeks of the study; HR-HPV detection decreased from 62% to 39%.

The risk of LR-HPV type persistence was higher in HIV-infected women compared to HIV-uninfected women. The significance of this observation is not known, but while HR-HPV types account for the majority of cervical cancers worldwide, it is possible that LR-HPV types may be more pathogenic in HIV-infected women, especially the types that were once considered “intermediate risk” types by IARC. Further studies are needed to clarify this issue.

Limitations of this study include a modest number of women enrolled, although each participant was sampled repeatedly, providing additional statistical power. The sampling method utilized could reduce the generalizability of the findings. Because most HIV-infected women were receiving ART, it was not possible to perform a direct comparison of women receiving ART versus women not receiving ART to determine the direct effect on reducing HPV persistence. As this study progresses, the effects of specific HIV-related variables (including the specific ART regimens/combinations in use) on HPV incidence, persistence, and type distribution will be evaluated.

## Conclusion

In conclusion, HPV persistence was examined in this longitudinal study of Kenyan women, approximately half of whom were HIV-infected, receiving effective ART, and had re-constituted immune systems based on satisfactory CD4 cell counts. Persistent detection of any HPV, HR-HPV, IARC HR-HPV, and LR-HPV types were all significantly higher in HIV-infected women compared to HIV-uninfected women. In addition to HIV infection, factors associated with an increased risk of HPV persistence included a higher number of lifetime sex partners. Factors associated with a decreased risk of HPV persistence included older age and being married. To prevent cervical cancer, a malignancy caused by persistent HR-HPV infection, it is important to study and understand the behavioral and specific immunological defects that remain in HIV-infected women receiving ART that allow HPV to persist. Further studies are also needed to determine the causes and significance of LR-HPV persistence in HIV-infected Kenyan women.

## Supplemental Material

Project_1_questionnaire – Supplemental material for Persistence of oncogenic and non-oncogenic human papillomavirus is associated with human immunodeficiency virus infection in Kenyan women Click here for additional data file. Supplemental material, Project_1_questionnaire for Persistence of oncogenic and non-oncogenic human papillomavirus is associated with human immunodeficiency virus infection in Kenyan women by Yan Tong, Philip Tonui, Aaron Ermel, Omenge Orang’o, Nelson Wong, Maina Titus, Stephen Kiptoo, Kapten Muthoka, Patrick J Loehrer and Darron R Brown in SAGE Open Medicine
