# Supplementary material for: Persistence of oncogenic and non-oncogenic human papillomavirus is associated with human immunodeficiency virus infection in Kenyan women
Source: SAGE Open Med. 2020 Jul 28;8:2050312120945138. doi: 10.1177/2050312120945138 (PMC7388102; doi:10.1177/2050312120945138)
Supplement: Project_1_questionnaire – Supplemental material for Persistence of oncogenic and non-oncogenic human papillomavirus is associated with human immunodeficiency virus infection in Kenyan women [file Project_1_questionnaire.pdf]

MODIFIABLE FACTORS PREDICTING PERSISTENCE OF ONCOGENIC HPV AND CERVICAL DYSPLASIA IN HIV INFECTED KENYAN WOMEN STUDY.

STUDY QUESTIONNAIRE

Participant study number________________ Participant Initials: _________

Date __ __/__ __/__ __ __ __

Visit (*tick one)*

Enrollment Visit		3 mo		6 mo		9 mo		1 yr	
		15 mo		18 mo		21 mo		2 yr	
		27 mo		30 mo		33 mo		3 yr	
		39 mo		42 mo		45 mo		4 yr	

Enrolment and Annual Visit: All questions

1. What is your HIV status? (*Tick one choice)*
   HIV positive□
   HIV negative □
   Unknown□

2. What is the HIV status of your partner? *(Tick one choice)*
   HIV positive□
   HIV negative □
   Unknown□

3. What was your age at first sexual encounter? ____________

4. Please indicate your number of live births______ and abortions/miscarriages_______

5. How many sexual partners have you had in the past 6 months? _______

Quarterly Visits

6. How many times in the last 3 months have you had sexual intercourse?
   _____

7. How many times in the past 3 months did you have sex using a condom? ______

8. What sexually transmitted infections have you had? *(Tick all that apply)*
   Chlamydia□ Trichomoniasis□ Gonorrhea□
   Herpes□ Bacterial vaginosis□ None□

Other□ ______________________

9. What family planning method do you currently use? *(Tick all that apply)*
   Contraceptive pills □ IUD □ Implant □ Patch□
   Injectable □ Condom □ None□
   Other □ ____________________

10. Do you have any other medical problems/illnesses do you have?

    Yes □ No □

    If yes, which ones?

    Diabetes □

    Hypertension □

    Other □

11. Are you using any other medication?
    Yes □ No □

    If yes, which ones?

    Prescription □ Over the counter□ Herbal□

12. What is your current smoking status? Please check what best describes your smoking.
    - I do not smoke□
    - Once or twice a month□
    - Once or twice a week□
    - Nearly every day□

13. How many hours per week are you in contact with indoor smoke from cooking or heating? ______

14. Before now, have you had any procedures performed due to abnormalities of the cervix?

Yes□ No□

15.VIA result:

Negative □

Positive □

HIV-infected participants, enrolment and annual visit:

(Study staff to abstract from the AMRS)

16. **WHO Stage:** 1□ 2 □ 3 □ 4 □ **Criteria:**

**Latest Test Results: (Please record date sample was drawn)**		
**Test**	**Result**	**Test Date**
CD4		/ /
Plasma Viral Load		/ /

17. Current HAART regimen Yes□ No□

□ NVP200/ZDV300/3TC/150<br>□TDF300mg/3TC300mg/EFV600mg<br>□ NVP200/D4T30/3TC150<br>□ 3TC150mg/ZDV300mg	□ 3TC300mg/TDF300mg<br>□ 3TC150mg/D4T30mg<br>□ Efavirenz600mg<br>□ Nevirapine200mg	□ Abacavir300mg<br>□ Lamivudine150mg<br>□ Zidovudine 300mg<br>□Aluvia**(kaletra)**200mgLPV /50mgrit	□ Raltergravir400mg<br>□Truvada***(Emtri200mg/TDF300)***<br>□Other:_______________ ______________________

Completed by: Sign ____

Name ____

Data entry by: Sign ____

Name ____
